# Two-dimensional Speckle Tracking Imaging in Cardiotoxicity Caused by Treatment of Breast Carcinoma with Anthracyclines

**DOI:** 10.7150/jca.98204

**Published:** 2024-09-09

**Authors:** Wenjuan Song, Xuejuan Ma, Yue Sun, Liping Liu, Ying Gu, Yue Zhao, Yujia Ye, Yu Wang

**Affiliations:** Department of Cardiology, The First Affiliated Hospital of Kunming Medical University, Kunming, 650032, Yunnan Province, China.

**Keywords:** routine echocardiography, two-dimensional speckle tracking imaging, anthracyclines, breast carcinoma, cardiotoxicity

## Abstract

**Objective:** This research was conducted to investigate the monitoring values of routine echocardiography (ECG) and two-dimensional speckle tracking imaging (2D-STI) in cardiotoxicity caused by the treatment of breast carcinoma with anthracyclines (ANTH).

**Methods:** 100 patients with breast carcinoma were selected and enrolled into normal group (n=53 cases) and abnormal group (47 cases) according to whether ECG was abnormal. Routine ECG and 2D-STI were employed for the detection, ECG- and 2D-STI-related parameters were compared, receiver operating characteristic (ROC) curves were drawn, and the clinical application values of monitoring methods for two groups were assessed.

**Results:** Before chemotherapy, no remarkable statistical difference was detected in routine ECG and 2D-STI parameters between normal and abnormal groups (*P*>0.05). After 6 cycles, E/V value of abnormal group was inferior to that of normal group ((0.93±0.16) vs (1.33±0.23). Besides, longitudinal peak strain (SRI) values of rear wall, front spacer, and rear spacer in abnormal group were inferior to those in normal group (*P*<0.05). Routine ECG combined with 2D-STI had the best predictive effect followed by 2D-STI and routine ECG.

**Conclusion:** To sum up, 2D-STI was a new method for assessing myocardial lesions and possessed significant early clinical monitoring values in cardiotoxicity caused by chemotherapy after the treatment of breast carcinoma with ANTH. It had higher clinical application values than routine ECG.

## Introduction

As one of the commonest malignant tumors in women worldwide, breast carcinoma poses some threats to patient's lives. Current clinical research focuses on the improvement of therapeutic effect on breast carcinoma and the reduction of mortality 1. At present, postoperative adjuvant chemotherapy is a widely recognized method for improving the survival and reducing the recurrence rate among patients with breast carcinoma 2. CMF regimen (cyclophosphamide+methotrexate+fluorouracil) is a classical chemotherapy that has long been adopted in clinical practice 3. However, some research demonstrated that the combined therapy of anthracyclines (ANTH) and chemotherapy was much more effective than routine chemotherapy 4,5.

ANTH refers to a series of antineoplastic drugs and antibiotics, such as adriamycin, daunorubicin, demethyloxygendaunorubicin, and aclacinomycin 6. In recent years, ANTH plays a vital role in treating multiple tumor diseases, such as breast carcinoma and acute leukemia. Adriamycin, epirubicin, and pirarubicinare mainly adopted to treat breast carcinoma. Although clinical ANTH shows significant therapeutic effects, they cause apparent toxic and side effects due to cytotoxicity, such as bone marrow supression, immune suppression, and cardiotoxicity 7. Cardiotoxicity is the severest and irreversible toxic and side effect that may threaten patients' lives, which is caused by limited chronic dose accumulation. In early treatment stage, ANTH causes different levels of myocardial damages 8. At present, the abnormalities among patients can't be timely detected by clinical cardiac function assessment, cardiac uhrasonography, and laboratory examination. Abnormalities suggest serious cardiac dysfunctions among patients, which should be clinically prevented mainly through cardiac monitoring for patients treated with ANTH as early as possible. Echocardiography (ECG) can be utilized to display cardiac anatomical structure and hemodynamics changes and assess cardiac functions. The main monitoring and assessment indicators for cardiotoxicity are ventricular diastolic and systolic functions, especially left ventricular ejection fraction (LVEF) 9. According to relevant research, routine ECG has some application values in the monitoring of cardiotoxicity after ANTH chemotherapy 10. To further improve the monitoring and assessment techniques of ECG, two-dimensional speckle tracking imaging (2D-STI) is proposed after the continuous exploration over the past years 11,12. It is a more advanced ECG technique that isn't affected by the angle dependence between sonic beam and the direction of ventricular wall motion in quantitative cardiac assessment. Local and general myocardial functions are evaluated by calculating the corresponding strains, strain rates, time to peak, and rotation and unwinding angles on the longitudinal, radial, and circumferential planes of cardiac motion 13,14. Nonetheless, there is no research into the application of routine ECG combined with 2D-STI in cardiotoxicity monitoring caused by the treatment of breast carcinoma with ANTH.

In this research, routine ECG and 2D-STI were employed to monitor cardiotoxicity caused by the treatment of breast carcinoma with ANTH. Relevant parameters were measured to analyze its predictive effectiveness in cardiac systolic dysfunction and its application values were assessed to provide a more effective and accurate assessment method for early monitoring of cardiotoxicity and improve survival rate.

## Research methods

### Subjects

100 patients diagnosed with breast carcinoma at oncology department of The First Affiliated Hospital of Kunming Medical University Cancer Hospital between February, 2023 and January, 2024 were selected as the research objects.

The inclusion criteria were listed below.

A. All patients were diagnosed for the first time without a history of malignant tumors.

B. All patients underwent radical surgery and postoperative ACT (cyclophosphamide+pirarubicin was adopted for 4 cycles), CAF (pirarubicin+cyclophosphamide+fluorouracil), or TE (epirubicin+docetaxel) as adjuvant chemotherapy methods.

The exclusion criteria were listed below.

A. Patients who suffered from severe heart diseases with abnormalities in ECG examination.

B. Patients who suffered from immune system diseases and diabetes.

C. Female patients who were during gestational period.

The statistical results showed all included patients were females aged between 20 and 70 with the average at 46.21±11.73. After receiving chemotherapy for 6 cycles, they all underwent ECG monitoring. According to whether there were abnormalities in ECG examination, they were divided into normal group (n=53 cases) and abnormal group (47 cases). 53 patients in normal group were aged between 21 and 70 with the average age at 47.33±11.32. 16 received ACT, 19 underwent CAF, and 18 were performed with TE. 47 patients in abnormal group were aged between 20 and 70 with the average age at 45.88±10.09. 13 received ACT, 14 underwent CAF, and 16 were performed with TE. No remarkable difference was detected in the general clinical data on patients in the two groups (*P*>0.05). Routine ECG and 2D-STI were employed to perform monitoring on the patients in the two groups. Relevant routine ECG parameters and 2D-STI strain parameters were then acquired, receiver operating characteristic (ROC) curves were drawn, and the clinical application values of the two monitoring methods were assessed.

All included patients had been informed of the research profile and signed informed consent forms. The implementation of this research had been approved by Hospital Medical Ethics Committee.

### ECG examination

Doppler ultrasound diagnosis instrument (Phillips, IE33) and S5-1 probe with the frequency between 2Hz and 4Hz were utilized for the examination on all included patients. ECG examination was carried out before and 6 cycles after chemotherapy. During the examination, patients were instructed to take left lateral position and stay calm. After left anterior chest was fully exposed, routine 2D ECG and color Doppler ultrasonography were implemented. The main parameters to be detected included left atrial diameter at end-diastole (LADD), left ventricular internal diameter at end-diastole (LVIDD), left ventricular internal diameter at end-systole (LVIDS), interventricular septal thickness at end-diastole (IVSD), left ventricular posterior wall diameter at end-diastole (LVPWD), and the ratio of bicuspid valve orifice at early-diastole (E) to peak flow velocity (A). Teichholtzwas adopted to measure and calculate LVEF.

### 2D-STI measurement

Based on ECG examination, ECG was connected and 2D-STI was utilized to acquire the dynamic images of apical four-chamber view, apical two-chamber view, and left ventricular long axial view in the following steps. When patients took breath steadily, 3 stable and consecutive cardiac cycles were intercepted and then uploaded to work station to generate region of interest (ROI). Then, ROI was appropriately adjusted to make it consistent with the thickness of left ventricular wall. After that, the relevant parameters of 18 segments of left ventricular long axis were acquired, including basal segment, middle segment, and longitudinal peak strain (SRI) values of apical front wall, rear wall, side wall, lower wall, front spacer, and rear spacer.

### Statistical methods

SPSS 22.0 was adopted for statistical analysis. Measurement data conforming to normal distribution were denote by x±s and the differences between groups were compared using one-factor analysis of variance. Pairwise comparison between groups was carried out using least significant difference-t (LSD-t) test. ROC curves were employed to analyze the sensitivity, specificity, positive and negative predictive values, and accuracy of the prediction of the injuries to cardiac systolic functions by ECG combined with 2D-STI parameters. *P*<0.05 revealed that the difference demonstrated statistical significance.

## Results

### Statistics on ECG abnormalities among 100 patients

**Figure 1:**
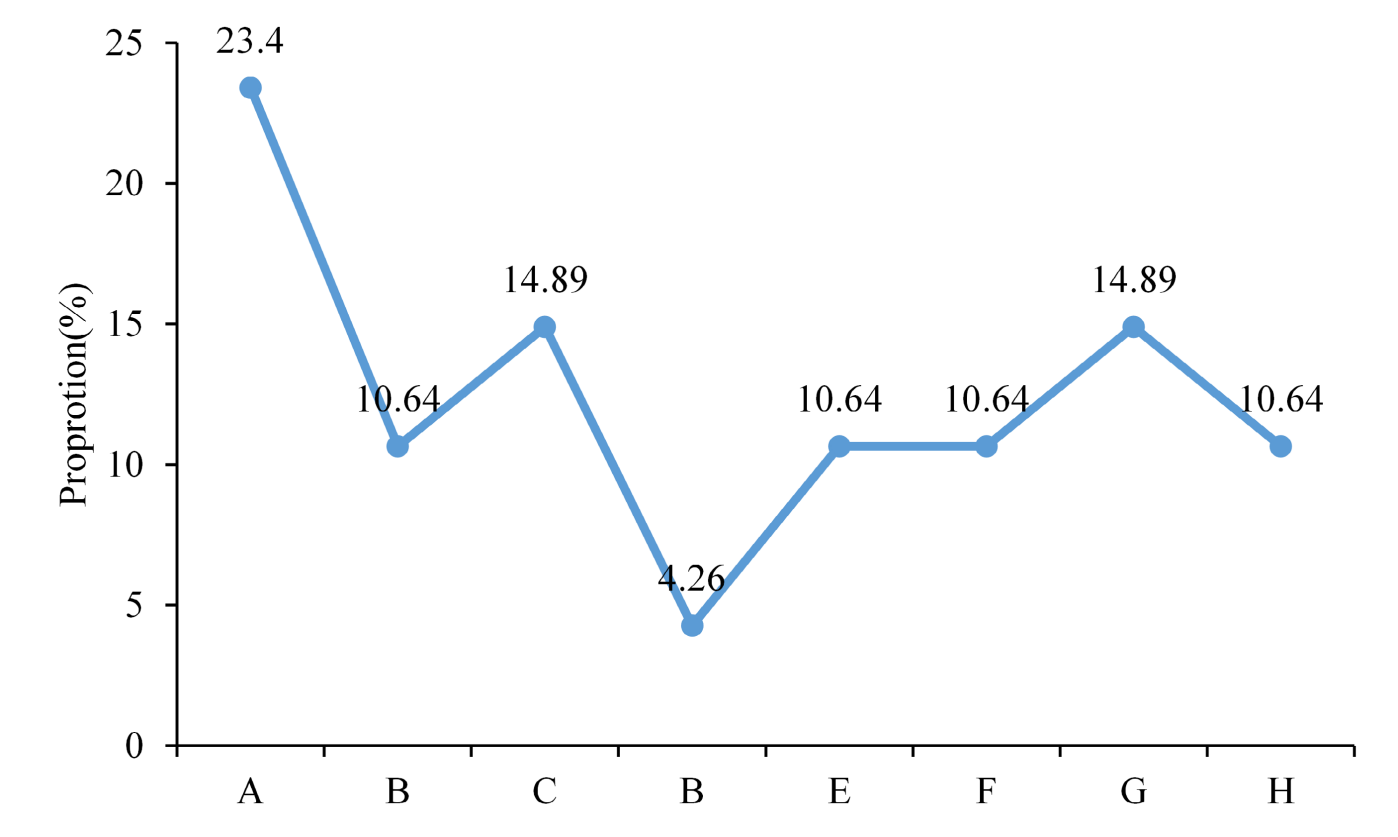
Statistics on ECG abnormalities (A. Premature ventricular beat. B. Horizontal ST segment with T wave inversion. C. Declivitous ST segment with T wave inversion. D. Horizontally extended ST segment. E. Left bundle-branch block. F. Atrial fibrillation. G. QRS wave with low voltage. H. Supraventricular tachycardia).

ECG was utilized to carry out cardiac examination on 100 patients 6 cycles after chemotherapy. ECG abnormalities were detected among 47 patients. There were 11 cases with premature ventricular beat, 5 with horizontal ST segment with T wave inversion, 7 with declivitous ST segment with T wave inversion, 2 with horizontally extended ST segment, 5 with left bundle-branch block, 5 with atrial fibrillation, 7 with QRS wave with low voltage, and 5 with supraventricular tachycardia (Figure 1). ECG indicators of the rest of patients were generally normal.

### Results of routine ECG examination

**Figure 2:**
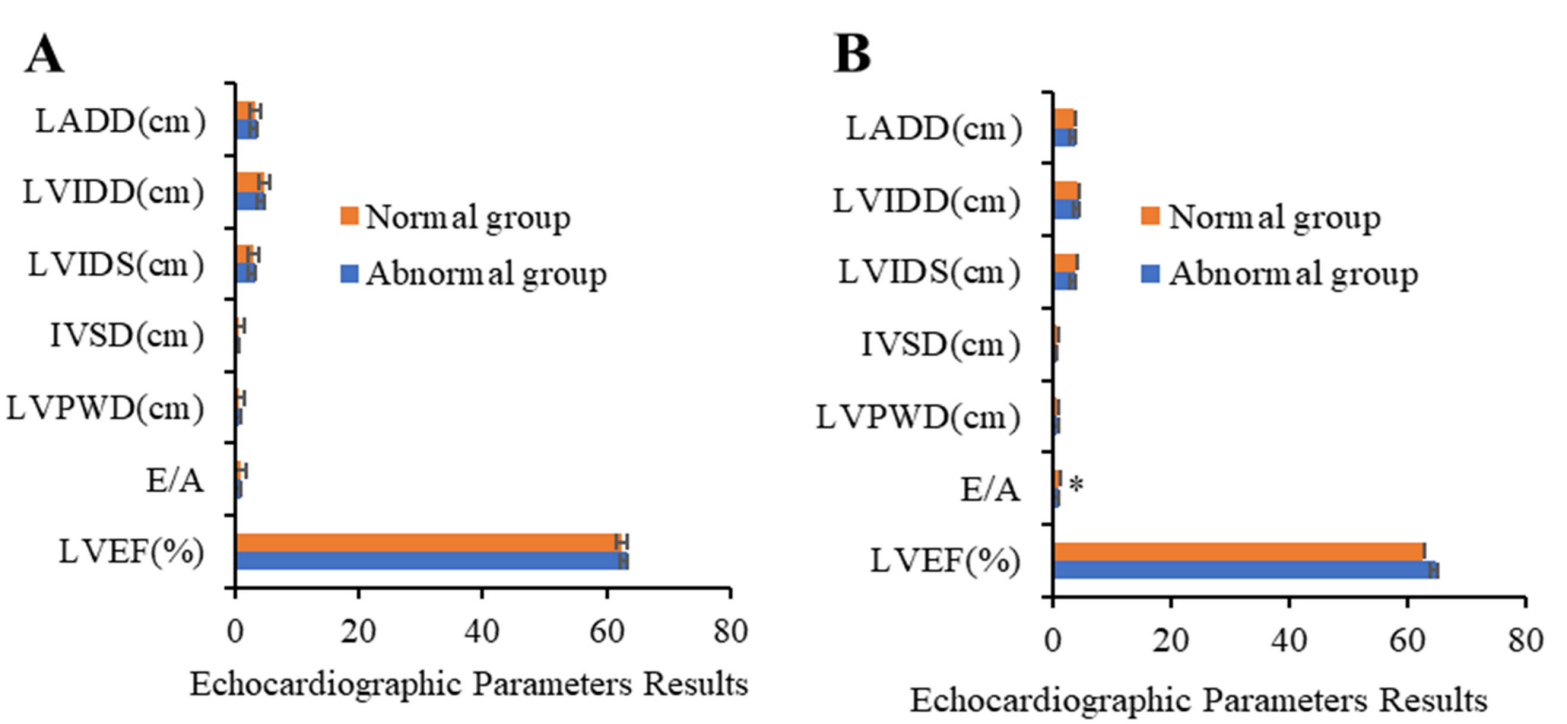
Results of ECG examination before and after chemotherapy (* suggested the differences between the two groups revealed statistical significance (*P*<0.05)).

The results of routine ECG examination on patients in the two groups before and after chemotherapy were compared and analyzed. It was suggested that no remarkable statistical differences were detected in LADD, LVIDS, IVSD, LVPWD, E/A, and LVEF between the two groups before chemotherapy (*P*>0.05). As presented in Figure 2-A, no remarkable statistical differences were detected in LADD, LVIDS, IVSD, LVPWD, and LVEF between the two groups 6 cycles after chemotherapy (*P*>0.05). E/A value of abnormal group was notably inferior to that of normal group (0.93±0.16 vs 1.33±0.23) (*P*<0.05) (Figure 2-B).

### Comparison of SRI parameters before and after chemotherapy

**Figure 3:**
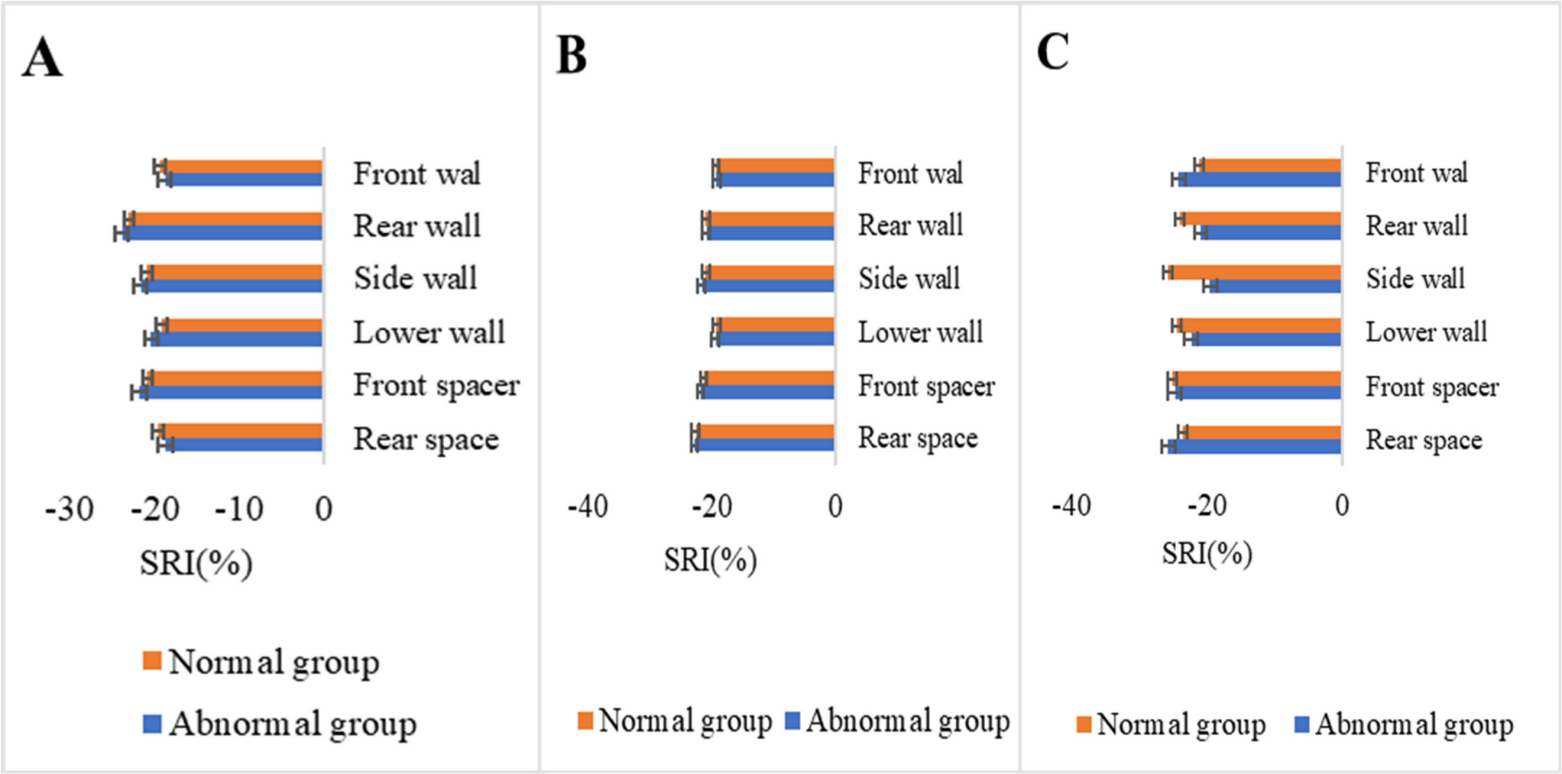
Comparison of SRI parameters before chemotherapy (A. Basal segment. B. Middle segment. C. Apical segment).

SRI values of basal segment, middle segment, and apical front wall, rear wall, side wall, lower wall, front spacer, and rear spacer of patients in normal and abnormal groups before chemotherapy were displayed in Figure 3. No remarkable statistical significance was detected in SRI values between the two groups (*P*<0.05).

**Figure 4:**
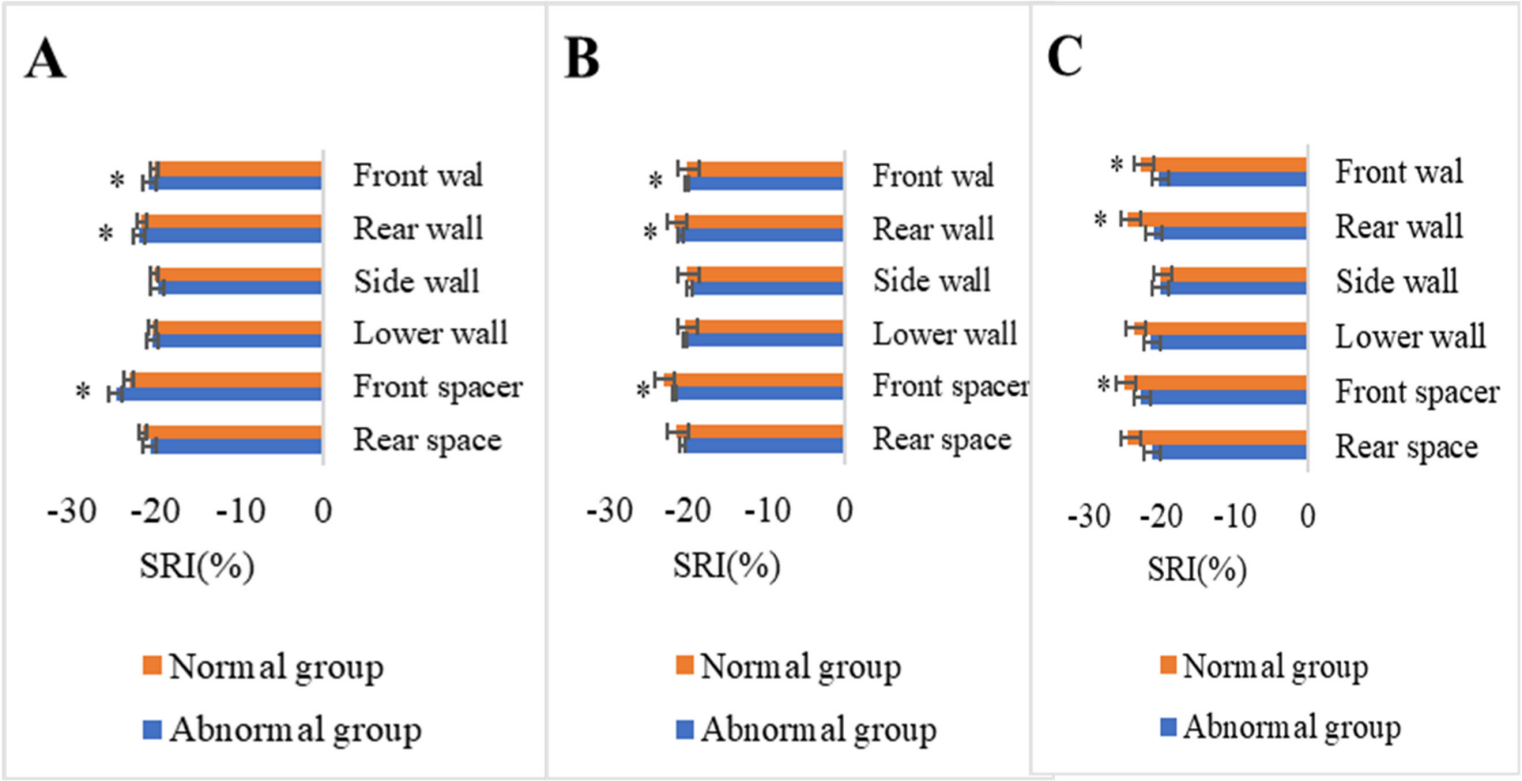
Comparison of SRI parameters after chemotherapy (A. Basal segment. B. Middle segment. C. Apical segment. * suggested the differences between the two groups revealed statistical significance (*P*<0.05)).

SRI values of basal segment, middle segment, and apical front wall, rear wall, side wall, lower wall, front spacer, and rear spacer of patients in normal and abnormal groups after chemotherapy were shown in Figure 4. No remarkable statistical significance was detected in longitudinal SRI values of left ventricular front wall, side wall, and lower wall between the two groups (*P*>0.05). SRI values of rear wall, front spacer, and rear spacer of patients in abnormal group declined versus those of patients in normal group (*P*<0.05).

### Analysis of forecasting ROC curves of various parameters for myocardial injury

**Figure 5:**
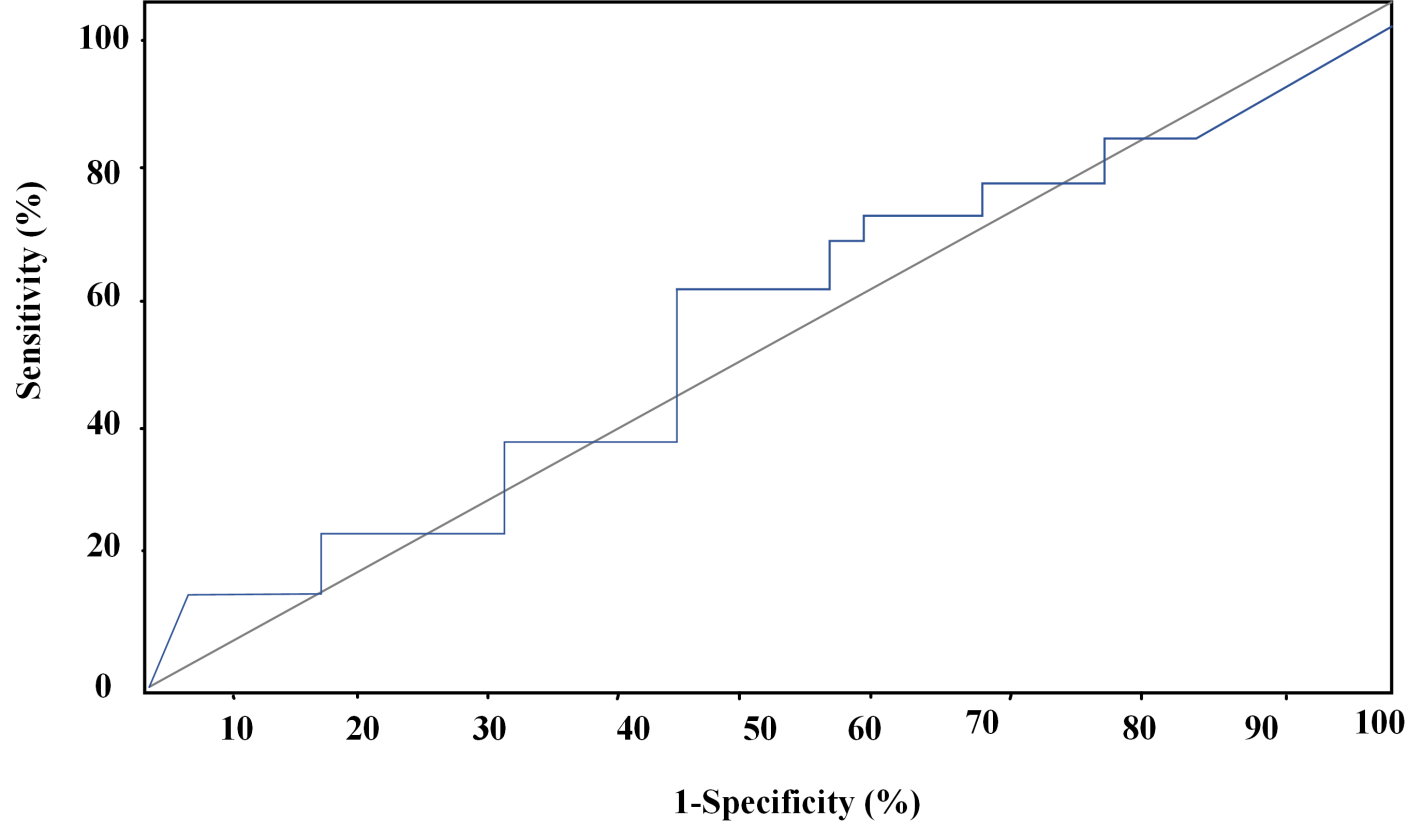
ROC curve of the prediction of myocardial injury by routine ECG.

In this research, forecasting ROC curves of routine ECG, 2D-STI, and the combined detection of routine ECG and 2D-STI for myocardial injury were drawn based on the results of troponin diagnosis. ROC forecasting curve of routine ECG was displayed in Figure 5. Area under the curve (AUC) of forecasting ROC curve of routine ECG for myocardial injury amounted to 0.65 6 cycles after chemotherapy and -40.02% was taken as the optimal critical value. The sensitivity, specificity, positive and negative predictive values, and accuracy of the prediction for cardiac systolic dysfunction amounted to 60.09%, 60.23%, 70.93%, 67.44%, and 78.56%, respectively.

**Figure 6:**
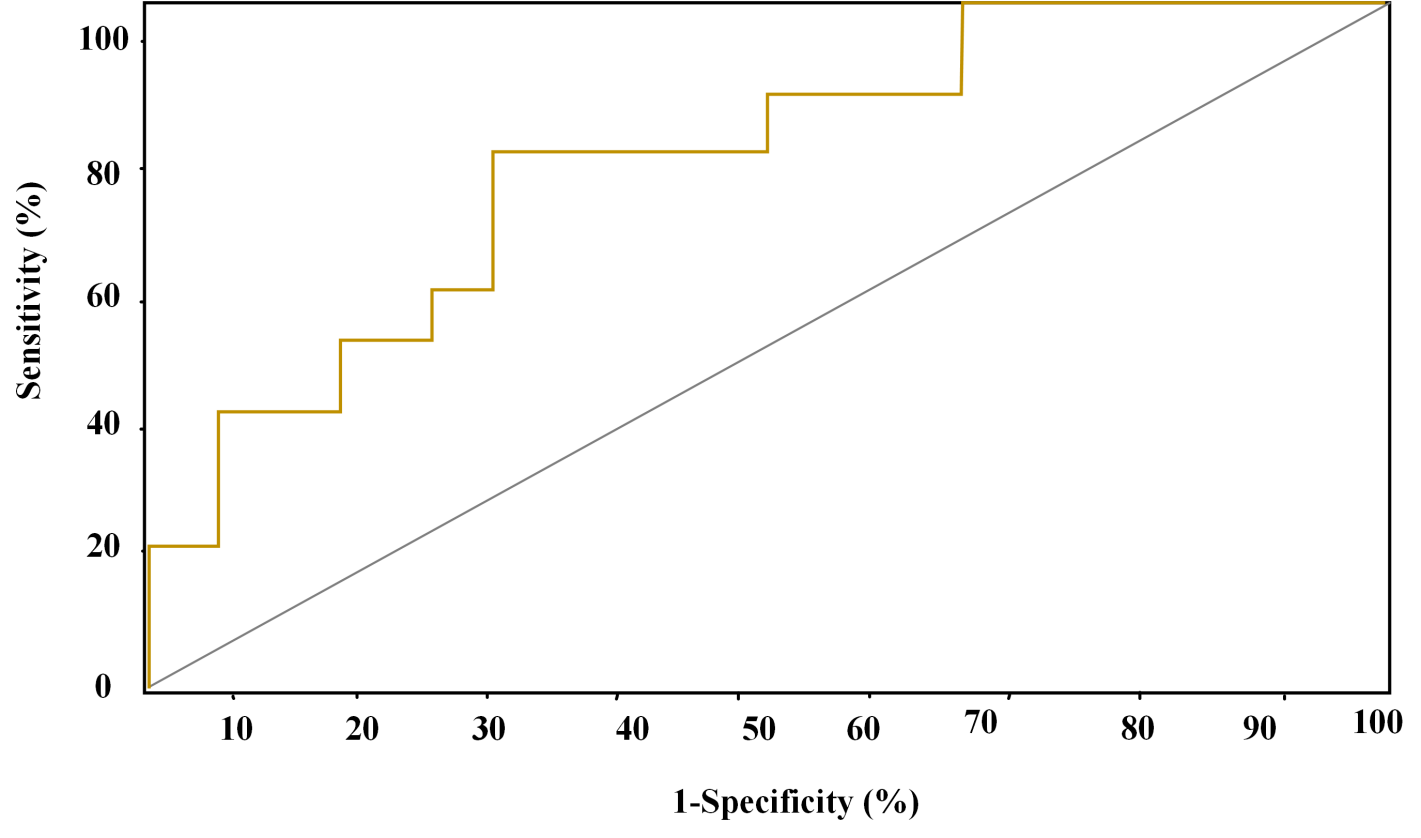
ROC curve of the prediction of myocardial injury by 2D-STI.

ROC forecasting curve of 2D-STIwas displayed in Figure 6. AUC of forecasting ROC curve of 2D-STI for myocardial injury amounted to 0.83 6 cycles after chemotherapy and 32.45% was taken as the optimal critical value. The sensitivity, specificity, positive and negative predictive values, and accuracy of the prediction for cardiac systolic dysfunction amounted to 87.56%, 80.45%, 90.45%, 82.54%, and 87.67%, respectively.

**Figure 7:**
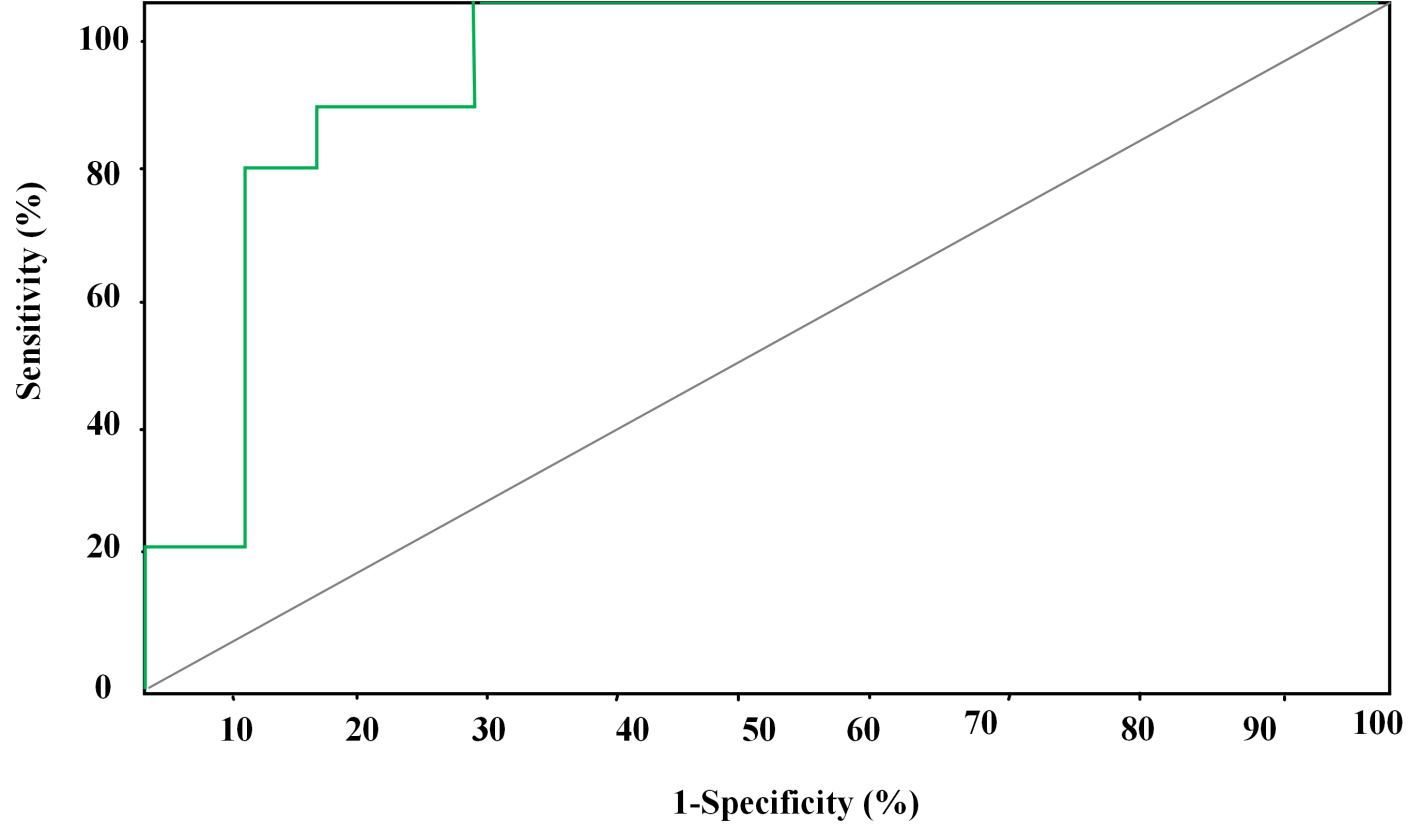
ROC curve of the prediction of myocardial injury by the combined detection of routine ECG and 2D-STI.

ROC forecasting curve of the combined detection of routine ECG and 2D-STIwas displayed in Figure 7. AUC of forecasting ROC curve of the combined detection for myocardial injury amounted to 0.98 6 cycles after chemotherapy and 14.34% was taken as the optimal critical value. The sensitivity, specificity, positive and negative predictive values, and accuracy of the prediction for cardiac systolic dysfunction amounted to 92.98%, 96.44%, 93.34%, 90.03%, and 94.76%, respectively.

**Figure 8:**
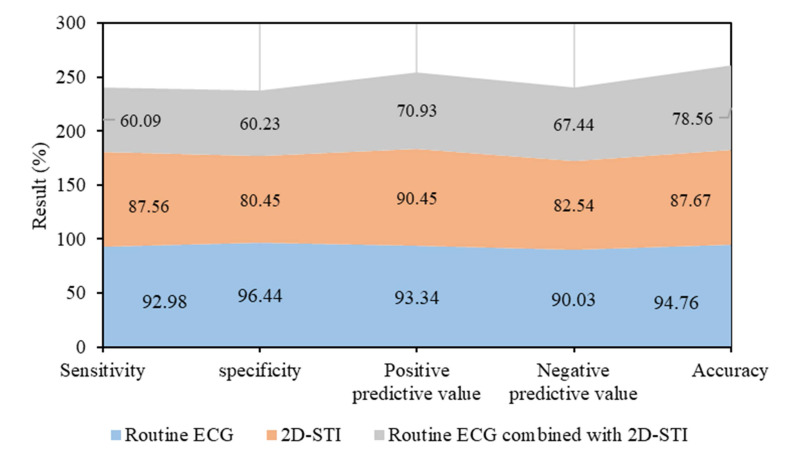
Comparison of the predictive effects of 3 detection methods.

According to the above results, the predictive effects of the 3 detection methods were compared (Figure 8). Routine ECG combined with 2D-STI had the best predictive effect followed by 2D-STI and routine ECG.

## Discussion

This study compared conventional electrocardiography (ECG) with 2D-speckle tracking imaging (2D-STI) in monitoring cardiotoxicity in breast cancer patients treated with anthracycline drugs. The study found significant differences in cardiac functional parameters between abnormal and normal groups before and after chemotherapy. After chemotherapy, patients in the abnormal group had significantly lower E/V ratio values compared to the normal group, and there was also a significant decrease in longitudinal peak strain (SRI) values of the posterior wall, anterior septum, and posterior septum. These results suggest that anthracycline drugs may cause myocardial dysfunction, particularly more significantly in patients with pre-existing cardiac dysfunction. Our findings are consistent with some studies in the existing literature 15, which also indicate that anthracycline drugs may induce cardiotoxicity and suggest that ECG and 2D-STI have potential diagnostic value in early detection. Previous research has shown that cardiotoxicity can lead to abnormalities in myocardial contraction and relaxation, which is consistent with our observations of decreased E/V ratio and SRI values 16, 17. The study indicates that conventional ECG and 2D-STI may complement each other in monitoring breast cancer patients treated with anthracycline drugs. While the differences between these methods before chemotherapy were not significant, the abnormal group showed clear signs of myocardial dysfunction in 2D-STI parameters after chemotherapy. This suggests that combining multiple imaging methods can enhance early diagnostic capabilities for cardiotoxicity, helping to adjust treatment plans promptly and improve patient outcomes.

Fatemeh *et al.* (2022) 18 pointed out that no left ventricular systolic and diastolic dysfunctions occurred among patients after chemotherapy with low-dose ANTH. As the number of research objects increased, follow-up duration was prolonged, and the dose of ANTH increased, it was confirmed that long use of low-dose ANTH caused the damages to left ventricular systolic and diastolic dysfunctions. Henriksen (2018) 19 also showed that chemotherapy with ANTH led to dose-related myocardial cell injury and death and left ventricular dysfunction. Minotti *et al.* (2022) 20 held that ANTH might result in diastolic dysfunction. The above research findings demonstrated that routine ECG possessed application values in monitoring cardiotoxicity during the treatment of breast carcinoma with ANTH. This study found no significant statistical differences in conventional electrocardiographic indicators (LADD, LVIDS, IVSD, LVPWD, E/A, LVEF) between the normal and abnormal groups before chemotherapy. However, after six cycles of chemotherapy, the E/V ratio in the abnormal group was significantly lower than that in the normal group.The decrease in the E/V ratio typically reflects abnormal left ventricular diastolic function, suggesting that anthracycline drugs may induce myocardial dysfunction during chemotherapy. This difference indicates that even when conventional electrocardiographic parameters do not capture significant abnormalities before chemotherapy, more sensitive parameters such as the E/V ratio can detect potential cardiac toxicity reactions earlier during treatment. The E/V ratio reflects early changes in left ventricular diastolic function, often detectable before myocardial damage occurs. This makes the E/V ratio capable of detecting myocardial dysfunction caused by anthracycline drugs and other chemotherapy agents earlier, even when traditional electrocardiographic parameters do not show obvious abnormalities. Compared to traditional electrocardiographic parameters like LVEF, which may be influenced by cardiac pumping function, the E/V ratio focuses more on myocardial diastolic function, making it more sensitive and specific for evaluating cardiac toxicity. The E/V ratio is a quantitative measurement that provides a more objective and accurate assessment of cardiac function.

In this research, 2D-STI was utilized to track SRI values of basal segmental, middle segmental, and apical front wall, rear wall, lower wall, side wall, and intraventricular wall of apical left ventricular long axis of normal and abnormal groups before and after chemotherapy with ANTH. It was revealed that no remarkable statistical significance was detected in above SRI values between the two groups before chemotherapy. SRI values of rear wall, front spacer, and rear spacer of abnormal group declined versus those of normal group 6 cycles after chemotherapy, which might be associated with the abnormalities in ST segment. In this research, ST abnormalities occurred among nearly 30% of included patients, which was higher than the proportions of patients with other ECG abnormalities. Non-specific changes in ST-T segment suggested that patients suffered from myocardial ischemia or necrosis 21. According to relevant research 22, ECG segmental motion abnormalities occurred in interventricular septum and left ventricular rear wall among patients during the changes in ST-T segment. Funabashi *et al.* (2022) 23 and Barisic (2022) 24 found that ECG showed no abnormal left ventricular wall motion when ST-T was normal. The above research conclusions provided some comprehensive supports for the research findings.

The predictive effectiveness of routine ECG, 2D-STI, and the combined detection of routine ECG and 2D-STI for myocardial injury was assessed based on the results of troponin diagnosis. ROC curves showed that the combined detection of routine ECG and 2D-STI possessed the best predictive effect followed by 2D-STI and routine ECG. Chen *et al.* (2022) 25 believed that 2D-STI ECG was more accurate and sensitive in monitoring the abnormalities of cardiac functions caused by ANTH therapy than routine ECG, which was consistent with the research finding that 2D-STI parameters were more superior to routine ECG parameters in predictive effectiveness. Tan *et al.* (2022) 26 also confirmed that specific speckle tracking ECG showed remarkable clinical application values in the early monitoring of toxicity-related myocardial injury among patients with breast carcinoma after chemotherapy with epirubicin. However, there was no research into the application of routine ECG parameters combined with 2D-STI parameters in myocardial injury. Hence, the monitoring effect of routine ECG combined with 2D-STI still needs to be further researched and verified to improve the credibility of this research.

## Conclusion

To conclude, 2D-STI possessed significant clinical application value in the early monitoring of cardiotoxicity caused by adjuvant chemotherapy of breast carcinoma with ANTH as a new assessment method for myocardial lesions. Its clinical application values were more significant than routine ECG. What's more, it was demonstrated that routine ECG parameters combined with 2D-STI parameters possessed superior application values in monitoring cardiotoxicity caused by ANTH. However, the above research finding needs to be further investigated and verified due to lack of the supports from relevant research. It was shown that reasonable use of ECG and 2D-STI in clinical practice had a brilliant application prospect for timely detection of cardiac dysfunction and the implementation of effective treatment plan.

## Ethics approval and consent to participate

Confirming that informed consent was obtained from all subjects and/or their legal guardian(s); this includes information regarding informed consent obtained from the study participant's parent or legal guardian for any participant below the age of consent. Confirmation that the guidelines outlined in the Declaration of Helsinki were followed.

The implementation of this work was approved by the ethics committee of The First Affiliated Hospital of Kunming Medical University (FAHKMU302).
